# IL‐2 therapy preferentially expands adoptively transferred donor‐specific Tregs improving skin allograft survival

**DOI:** 10.1111/ajt.15306

**Published:** 2019-03-15

**Authors:** Kulachelvy Ratnasothy, Jacintha Jacob, Sim Tung, Dominic Boardman, Robert Ian Lechler, Alberto Sanchez-Fueyo, Marc Martinez‐Llordella, Giovanna Lombardi

**Affiliations:** ^1^ MRC Centre for Transplantation Peter Gorer Department of Immunobiology Faculty of Life Sciences & Medicine King's College London London UK; ^2^ Department of Inflammation Biology MRC Centre for Transplantation Institute of Liver Studies Faculty of Life Sciences & Medicine King's College London London UK

**Keywords:** antigen presentation/recognition, basic (laboratory) research/science, graft survival, immune regulation, immunobiology, immunosuppression/immune modulation, organ transplantation in general, T cell biology, tolerance, translational research/science

## Abstract

Regulatory T cells (Tregs) have unique immunosuppressive properties and are essential to ensure effective immunoregulation. In animal models, Tregs have been shown to prevent autoimmune disorders and establish transplantation tolerance. Therefore, the prospect of harnessing Tregs, either by increasing their frequency or by conferring allospecificity, has prompted a growing interest in the development of immunotherapies. Here, employing a well‐established skin transplant model with a single major histocompatibility complex mismatch, we compared the therapeutic efficacy of adoptively transfer Treg with or without donor specificity and the administration of IL‐2 to promote in vivo expansion of Treg. We showed that IL‐2 treatment preferentially enhances the proliferation of the allospecific Tregs adoptively transferred in an antigen‐dependent manner. In addition, donor‐specific Tregs significantly increased the expression of regulatory‐related marker, such as CTLA4 and inducible costimulator (ICOS), in the skin allograft and draining lymph nodes compared to endogenous and polyclonal transferred Tregs. Importantly, by combining IL‐2 with donor‐specific Tregs, but not with polyclonal Tregs, a synergistic effect in prolonging skin allograft survival was observed. Altogether, our data suggest that this combination therapy could provide the appropriate conditions to enhance the immunoregulation of alloimmune responses in clinical transplantation.

## Abstract 2

Combining adoptive transfer of donor‐specific Tregs with low dose IL‐2 has a synergistic effect in prolonging MHC‐class I–mismatched skin transplant survival due to antigen‐driven Treg expansion.

**Abbreviations**
APC: antigen presenting cells
CNIs: calcineurin inhibitors
GvHD: graft‐versus‐host disease
IL-2: interleukin-2
IL‐2c: interleukin‐2 complexes
IS: immunosuppression
SEM: standard error of the mean
TCR: T cell receptor
Teffs: effector T cells
Tregs: regulatory T cells

## 1 INTRODUCTION

CD4^+^CD25^+^FoxP3^+^ regulatory T cells (Tregs) play a critical role in various immunological processes, and are responsible for the induction and maintenance of peripheral tolerance to both self and foreign antigens. In preclinical models, adoptive transfer of Tregs has been exploited to prevent autoimmune diseases and to promote transplantation tolerance by modulating the balance between pathogenic and regulatory immune responses.^1^ The clinical application of ex vivo expanded Tregs has been initially developed to inhibit graft‐versus‐host disease (GvHD) following stem cell transplantation and to prevent disease progression in Type 1 diabetes.^2^, ^3^, ^4^ More recently, the first clinical trials in renal transplant recipients have been published demonstrating that the injection of polyclonal expanded Tregs shortly after transplantation is safe and feasible.^5^, ^6^ Although, we have observed similar results in the completed clinical trials in kidney (the ONE Study) and liver (ThRIL) transplantation,^7^ the clinical efficacy of Treg transfer in promoting transplantation tolerance remains unproven.

Linked to the increased understanding about the mechanisms of graft rejection, we and others have demonstrated in preclinical models of transplantation that the adoptive transfer of murine Tregs with donor specificity was superior in promoting allograft survival compared to polyclonal Tregs.^8^, ^9^ We have extended these observations to human Tregs by showing in a humanized mouse model of human skin transplantation that Tregs generated by stimulation with donor antigen presenting cells (APC) were superior in protecting from skin graft damage compared to polyclonally expanded Tregs.^10^, ^11^ Although new trials are currently testing the applicability of donor‐specific Tregs in kidney and liver transplantation, the general view is that the exclusive transfer of Tregs alone is unlikely to be sufficient to induce transplantation tolerance and therefore combination therapies are required.

Interleukin‐2 (IL‐2) is essential for the optimal development, survival, and function of Tregs. In contrast to effector T cells (Teffs), resting Tregs constitutively express the high‐affinity IL‐2Rα (CD25), which makes them highly sensitive to very low doses of IL‐2. Binding to the IL‐2 receptor complex initiates sequential signaling events, including phosphorylation of STAT5, that result in transcriptional activation of immunoregulatory genes such as FOXP3 and CD25 itself, and prosurvival genes such as MCL1 and BCL2. In animal models of skin transplantation, lupus nephritis, and lung inflammation it has been shown that the administration of either low‐dose IL‐2 or rIL‐2/anti‐IL‐2 complexes (IL‐2c) increased the endogenous pool of Tregs and improved disease outcome.^12^, ^13^, ^14^ We have recently published that although calcineurin inhibitors (CNIs) markedly disrupt Treg homeostasis and have a negative impact on the most highly immunosuppressive Treg subsets, provision of exogenous IL‐2 reversed these effects and synergized with CNIs in neutralizing effector T cell responses and promoting allograft survival.^15^ In the clinic, increasing studies in patients with autoimmunity or GvHD have shown that low‐dose IL‐2 safely expands endogenous Tregs providing significant therapeutic benefits.^16^, ^17^, ^18^, ^19^^.^

In this study, we compared the efficacy of the different strategies proposed to modulate the Treg pool that can be translated into the clinical setting. Employing a very well‐established skin transplant model in which the skin is mismatched with the recipient for one single major histocompatibility complex class I molecule (K^d^), we demonstrated that IL‐2 treatment preferentially expands adoptively transferred in vitro generated donor‐specific Tregs leading to a synergist effect in extending graft survival.

## 2 MATERIALS AND METHODS

### 2.1 Mice

BL/6 (H‐2^b^) and CBA (H‐2^k^) mice aged 6‐8 weeks old were purchased from Charles River Laboratories. BL/6 Kd (BL/6 transgenic for K^d^), TCR75 (T cell receptor [TCR]‐transgenic mice recognizing Kd peptide H‐2A^b^), CD45.1 congenic mice on a BL/6 background were bred and maintained in the Biological Services Unit (New Hunt House) of King's College London. All animal experiments were performed according to UK Home Office.

### 2.2 Skin transplantation and treatments

Skin transplants were performed and monitored as described previously.^8^ In humans, low doses of rIL‐2 (0.5‐3 million IU/m2 day) increases the proportion and absolute number of circulating Tregs more than 2‐fold,^20^, ^21^ but in mice this increase is observed only following administration of rIL‐2/anti–IL‐2 antibody complexes (IL‐2c).^15^ IL‐2c was prepared by incubating 1 μg recombinant mouse IL2 (eBioscience) with 9 μg of functional grade purified antimouse IL‐2 (clone JES6‐1A12, eBioscience) for 30 minutes at 37°C, and it was injected intraperitoneally on days 0, 1, 2, and 3 posttransplant. For CD8 depletion, anti‐CD8 antibody (clone YTS 169, 250 μg/mice) was injected intraperitoneally day ‐1, +1, +7 posttransplant.

### 2.3 Generation and maintenance of CD4+CD25+ Treg lines

Previously generated autoreactive and indirect allospecificity Treg lines were used.^8^, ^9^ Briefly, B6‐S Tregs were generated by repeated stimulation with autologous BL/6 DCs and B6‐K^d^ Tregs were obtained after transduction with a TCR specific for K^d^ peptide presented by H‐2A^b^. Treg lines were maintained as described previously.^8^

### 2.4 Statistical analysis

The statistical analysis was performed by using GraphPad Prism 6 software (GraphPad Software Inc). Student's *t* test was used for comparison between two groups and analysis of variance with Tukey's post hoc correction for pairwise comparisons was used to compare more than 2 groups (*P < .05, **P < .01, ***P < .001 and ****P < .0001). Mean ± standard error of the mean (SEM) was routinely used.

## 3 RESULTS

### 3.1 TCR‐transduced Tregs are highly specific for the K^d^ alloantigen

**Figure 1:**
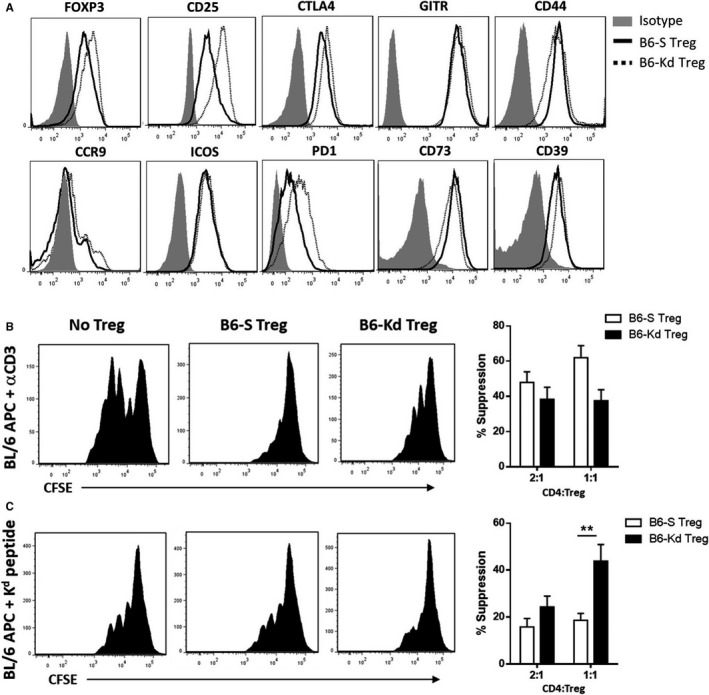
T cell receptor‐transduced Tregs are highly specific for allogenic K^d^ antigens. A, Surface expression for CD25, GITR, CD44, CCR9, ICOS, PD1, CD73, and CD39 as well as intracellular FOXP3 and CTLA4 on the two Treg lines was determined by flow cytometry (representative staining of > 3 performed). B‐C, Representative histograms of the division of CFSE‐labeled effector BL/6 CD4^+^ T cells 3 days after coculture with BL/6 APC plus anti‐CD3 (B) or BL/6 APC plus K^d^ peptides (C) in the presence or not of the two Treg lines (left panels). The percentages of suppression capacity at 2:1 and 1:1 ratio (APC:Treg) is shown in the right panel. Results represent mean ± standard error of the mean of three independent experiments. APC, antigen presenting cells; BL/6, C57BL/6; CCR9, C‐C chemokine receptor type 9; CFSE, carboxyfluorescein succinimidyl ester; CTLA4, cytotoxic T‐lymphocyte‐associated protein 4; FOXP3, Forkhead box p3+; GITR, glucocorticoid‐induced tumor necrosis factor receptor; ICOS, inducible costimulator; PD1, programmed cell death protein 1; Treg, regulatory T cell. **P < .01

To assess the effect of combining adoptive transfer of in vitro expanded Tregs with IL‐2 therapy, we employed two different Treg lines that we have reported previously,^8^, ^9^ one with self‐specificity (B6‐S Tregs) and the other with indirect allospecificity for K^d^ peptides presented by H‐2A^b^ (B6‐K^d^ Tregs). When subjected to flow cytometric analysis, both cell lines showed similar expression of markers related to regulatory function (Figure 1A). Next, the function of the two Treg lines was evaluated by measuring the suppressive effect on the proliferation of CFSE‐labeled effector BL/6 CD4^+^ T cells cocultured in the presence of autologous APC and anti‐CD3 (Figure 1B). Polyclonal proliferation of responder T cells was inhibited in a dose‐dependent manner and the percentages of suppression were comparable between the two Treg lines. The suppression of K^d^‐specific responses by the Treg lines was assessed by coculturing BL/6 APC pulsed with a K^d^ peptide and CFSE‐labeled BL/6 RAG‐/‐TCR75 CD4^+^ T cells, derived from TCR‐transgenic mice specific for K^d^ and restricted by A^b^ (Figure 1C). B6‐K^d^ Tregs showed an increased suppressive activity compared to the self‐reactive B6‐S Tregs in the inhibition of TCR75 T cell proliferation, further confirming the antigen‐specificity of the B6‐K^d^ Tregs.

### 3.2 Combining K^d^‐specific Tregs with IL‐2 therapy showed a synergistic effect in prolonging skin allograft survival

**Figure 2:**
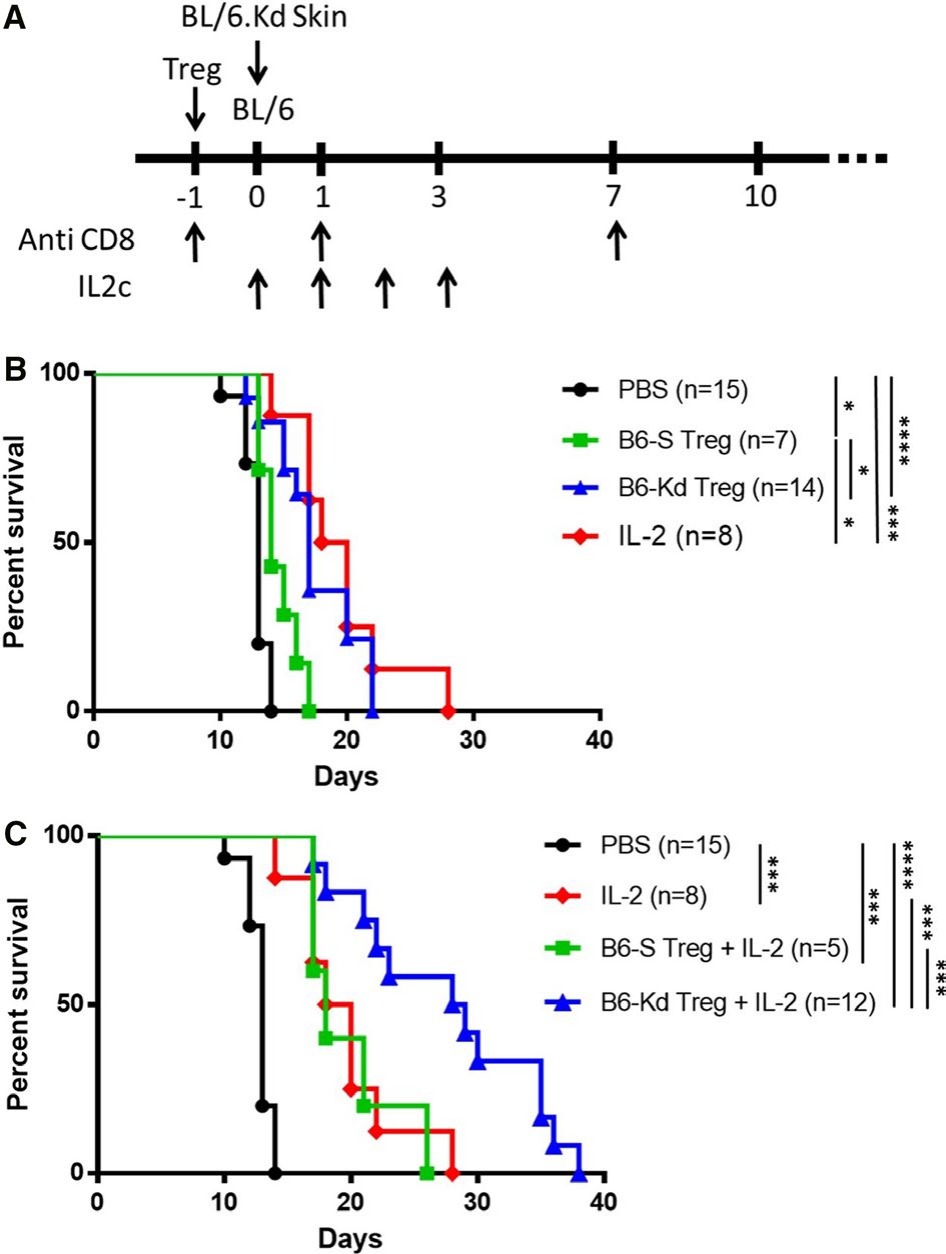
K^d^‐specific Tregs synergizes with IL‐2 therapy in prolonging skin allograft survival. A, Schema of the allogeneic BL/6.K^d^ skin transplant model. B‐C, Comparison of graft survival in mice with no cell transfer (PBS), B6‐S and B6‐K^d^ Tregs without (B) or with combination of IL‐2c treatment (C), as described in Materials and Methods. Results represent mean ± standard error of the mean of four independent experiments. B6‐Kd, Kd‐specific BL/6; B6‐S, Self‐specific BL/6; IL‐2, interleukin‐2; IL‐2c, interleukin‐2 complexes; Treg, regulatory T cell. *P < .05, ***P < .001, ****P < .0001 [Color figure can be viewed at wileyonlinelibrary.com]

Having shown that B6‐S and B6‐K^d^ Treg lines were phenotypically similar and suppressive in vitro, we compared their immunoregulatory function to the effect of endogenous Treg expansion caused by IL‐2 therapy in a transplant model where BL/6 mice were transplanted with skins derived from BL/6.K^d^ donors. CD8 depletion was performed by anti‐CD8 antibody injection on day ‐1, 1 and 7 after transplantation to inhibit the direct allospecific response to intact K^d^ molecules, as previously published.^8^ Recipient mice were treated with 5x10^6^ self‐specific B6‐S or donor‐specific B6‐K^d^ Tregs the day before transplant, or by IL‐2c injection from day 0 to day 3 following engraftment (Figure 2A). Although the self‐reactive Tregs were able to extend allograft survival only slightly (median: 13 vs 14 days), K^d^‐specific Tregs prolonged the median skin survival from 13 to 17 days (Figure 2B). Similarly, IL‐2c treatment showed significant prolongation of skin allograft survival (median 19 days).

Next, Treg adoptive transfer was combined with injection of IL‐2 to evaluate whether increased prolongation of skin transplant survival could be achieved (Figure 2C). Although combining IL‐2c treatment with self‐reactive Tregs did not increase the effect observed with IL‐2c alone, the combination of K^d^‐specific Tregs and IL‐2c prolonged allograft survival up to a median of 29 days. Altogether, these results demonstrate the advantage of combining Treg therapy with IL‐2 administration when antigen‐specific cells were used.

### 3.3 IL‐2c amplifies the number of adoptive transferred K^d^‐specific Tregs after skin transplantation

**Figure 3:**
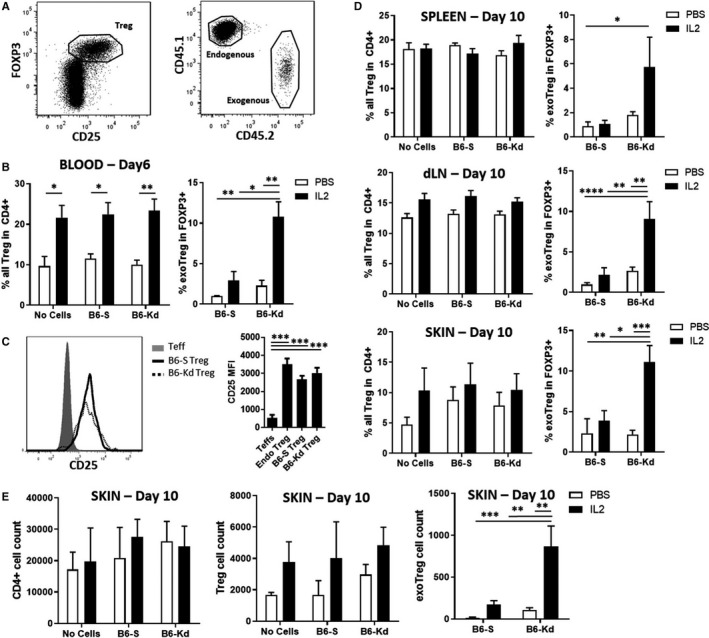
IL‐2c amplifies the number of adoptive transferred K^d^‐specific Tregs after skin transplantation. Tregs from different tissues were analyzed 6 and 10 days after BL/6‐K^d^ skin transplant to BL/6 mice receiving no cells, B6‐S or B6‐K^d^ Tregs in combination or not with IL‐2c. A, Representative dot plot of dLN CD4^+^ T cells based on CD25 and FOXP3 (left) and distribution of endogenous and exogenous Tregs based on CD45.1 and CD45.2 congenic markers (right). B, Percentage of total circulating Tregs among CD4^+^ T cells (left panel) and self‐ or donor‐specific Tregs among FOXP3 + cells (right panel) 6 days after transplantation in the presence of IL‐2c (black bar) or not (white bar). C, CD25 expression of blood Teffs, endogenous, B6‐S and B6‐K^d^ Tregs 6 days after transplantation. D, Percentages of total Tregs among CD4^+^ T cells (left panel) and self‐ or donor‐specific Tregs among FOXP3 + cells (right panel) 6 days after transplantation in the presence of IL‐2c (black bar) or not (white bar) in spleen, dLN and skin tissue. E, Cell count from skin allograft (0.5 cm^2^) of CD4 + (left panel), total Tregs (middle panel) and exogenous Tregs (right panel) 10 days after transplantation. Results represent mean ± SEM of at least three independent experiments. B6‐Kd, Kd‐specific BL/6; B6‐S, Self‐specific BL/6; dLN, draining lymph nodes; FOXP3, Forkhead box p3^+^; IL‐2, interleukin‐2; IL‐2c, interleukin‐2 complexes; PBS, phosphate‐buffered saline; Teff, effector T cells; Treg, regulatory T cell. *P < .05, **P < .01, ***P < .001, ****P < .0001

To assess the mechanisms behind the coordinated effect of IL‐2 therapy and B6‐K^d^ Treg combination, we analyzed the transferred (CD45.2) and endogenous (CD45.1) Tregs in different tissues at 6 and 10 days following skin transplantation in the same model described previously (Figure 3A). The analysis of blood samples 6 days after transplantation showed a significant increase in the total FOXP3^+^CD25^+^ Treg pool after IL‐2c treatment independently of adoptive transfer of Tregs (Figure 3B, left panel). However, when the percentages of transferred Tregs were evaluated, preferential expansion of the B6‐K^d^ Tregs compared to B6‐S, particularly after IL‐2c treatment, was observed (Figure 3B, right panel). CD25 expression was similar between the two Treg lines, suggesting that the distinctive expansion of B6‐K^d^ Tregs was not due to differential sensitivity to IL‐2 (Figure 3C).

The same analysis of Tregs was performed in the spleen, draining lymph nodes and skin on day 10 after transplantation. The frequencies of total Tregs were similar between all groups, indicating that the transferred Tregs did not alter the global distribution of Tregs among CD4^+^ T cells (Figure 3D, left panels). However, the analysis of adoptively transferred Treg lines showed a significant expansion of the K^d^‐specific Tregs in all the tissues when Treg therapy was combined with IL‐2c treatment (Figure 3D, right panels). Although the total number of CD4^+^ T cell and Tregs in the different tissues was not significantly increased by the early IL‐2c treatment, the absolute cell counts of K^d^‐specific Tregs were preferentially augmented in the skin allograft (Figure 3E & S1A).

### 3.4 Characterization of adoptively transferred Tregs

**Figure 4:**
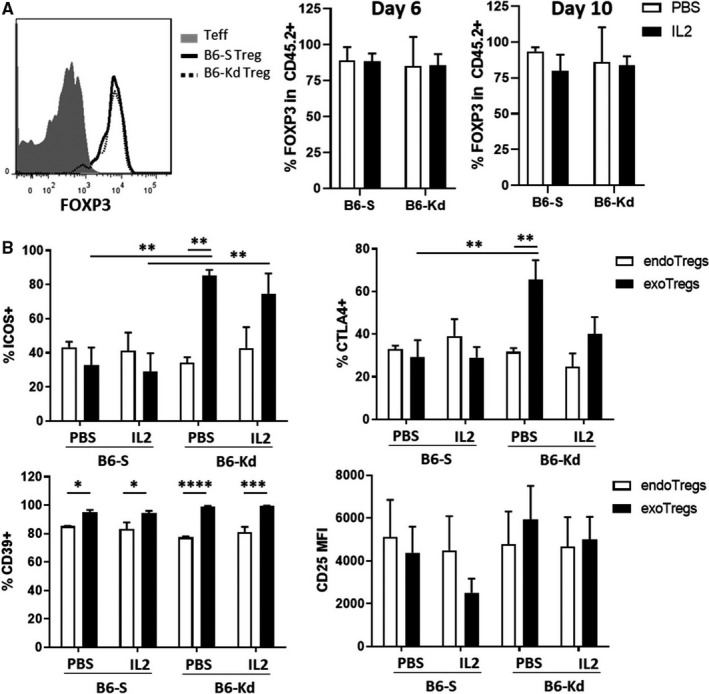
Characterization of adoptive transferred Tregs in skin transplant model. Tregs from different tissues were analyzed 6 or 10 days after BL/6‐K^d^ skin transplant to BL/6 mice receiving no cells, B6‐S or B6‐K^d^ Tregs in combination or not with IL‐2c. A, Representative FOXP3 expression histogram of circulating CD4 + T cells (left) and frequency of FOXP3 + among transferred CD45.2 + cells in blood 6 and 10 days after skin transplant (right). B, Frequency of ICOS+, CTLA4 + , CD39 +, and CD25 mean fluorescence intensity (MFI) on endogenous (CD45.1) and exogenous transferred (CD45.2) Tregs in draining lymph nodes 10 days after transplantation. Results represent mean ± standard error of the mean of two independent experiments. B6‐Kd, Kd‐specific BL/6; B6‐S, Self‐specific BL/6; CTLA4, cytotoxic T‐lymphocyte‐associated protein 4; FOXP3, Forkhead box p3+; ICOS, inducible costimulator; IL‐2, interleukin‐2; IL‐2c, interleukin‐2 complexes; PBS, phosphate‐buffered saline; Tregs, regulatory T cells. *P < .05, **P < .01, ***P < .001, ****P < .0001

The phenotypic stability of adoptively transferred Tregs was assessed by analyzing the percentage of FOXP3^+^ cells among the exogenous CD4^+^CD45.2^+^ cells. Sequential blood samples from day 6 and day 10 after skin transplantation revealed that the frequency of FOXP3^+^ cells within the adoptively transferred Tregs was maintained and this occurred independently of the IL‐2c treatment (Figure 4A). Similarly, Treg lineage preservation was observed in the spleen, draining lymph nodes and skin allograft at day 10 after transplantation (Figure S2A).

To further assess the functional characteristics of the two transferred Treg lines, the expression of several Treg‐related markers was investigated on endogenous and exogenous Tregs at day 10 after skin transplantation. In the draining lymph nodes, donor‐specific B6‐K^d^ Tregs showed an increased expression of CTLA4 and inducible costimulator compared to the endogenous and the self‐specific Tregs (Figure 4B); whereas no differences between the Treg lines were observed in the other tissues (Figure S2B). Although IL‐2c treatment augmented CD39 expression particularly in B6‐K^d^ Tregs (Figures 4B & S2B), no other major phenotypic effects were observed with the IL‐2 therapy, suggesting that the prolonged allograft survival in the combination therapy was due to the increased frequency of donor‐specific Tregs in the inflamed tissues.

### 3.5 Treg expansion during IL‐2c therapy is enhanced by antigen recognition

**Figure 5:**
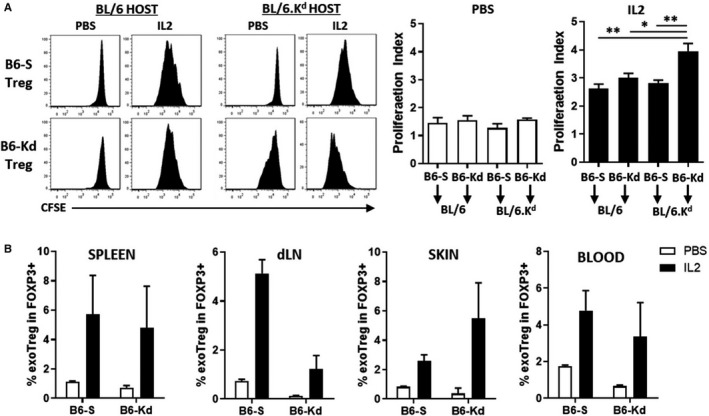
IL‐2c amplifies the number of adoptive transferred K^d^‐specific Tregs after skin transplantation. A, CFSE‐labeled B6‐S or B6‐K^d^ Tregs were transferred into BL/6 or BL/6K^d^ mice in combination or not with IL‐2c. Cell proliferation of transferred Tregs was assessed 3 days after injection by CFSE dilution (left panels) and their proliferation index was calculated using FlowJo V7 Proliferation Tool (right). B, Percentages of transferred B6‐S or B6‐K^d^ Tregs among FOXP3 + cells were analyzed from different tissues 10 days after CBA skin transplant to BL/6 mice in the presence (black bars) or not (white bars) of IL‐2c. Results represent mean ± standard error of the mean from same experiment. B6‐Kd, Kd‐specific BL/6; B6‐S, Self‐specific BL/6; CFSE, carboxyfluorescein succinimidyl ester; FOXP3, Forkhead box p3+; IL‐2, interleukin‐2; IL‐2c, interleukin‐2 complexes; PBS, phosphate‐buffered saline; Treg, regulatory T cell. *P < .05, **P <.01

To determine the extent to which antigen‐specific recognition modulates the expansion of Treg during IL‐2 treatment, CFSE‐labeled B6‐S and B6‐K^d^ Tregs were transferred to BL/6 or BL/6.K^d^ hosts. Mice were then treated with or without daily IL‐2c and Treg proliferation was evaluated 3 days after transfer (Figure 5A). The proliferation index of both Treg lines in BL/6 host mice was similar independent of IL‐2 treatment (Figure 5A, right panels). In BL/6.K^d^ hosts, however, although the proliferation of adoptively transferred Tregs was comparable in the absence of IL‐2, the expansion of K^d^‐specific Tregs was significantly higher after IL‐2 treatment. These data suggest that antigen recognition by Tregs is necessary for the synergistic effects of IL‐2 therapy to be seen. This was further confirmed by transferring B6‐S and B6‐K^d^ Treg lines to BL/6 mice transplanted with third‐party mismatched CBA donor skin (H‐2k). Following the same treatment regimen described in Figure 2, the distribution of adoptively transferred Tregs was analyzed on day 10 after transplantation in the spleen, draining lymph nodes, skin, and blood (Figure 5B). The frequencies of the transferred Treg lines among the whole Treg pool increased in a similar proportion by IL‐2 treatment, indicating comparable proliferative capacities against the third‐party allograft.

## 4 DISCUSSION

Treg homeostasis is highly dependent on the availability of IL‐2, which controls their survival and proliferation through different transcriptional programmes.^22^ Low‐dose IL‐2 therapy has shown in multiple animal models and clinical settings to induce the expansion of the Treg pool up to 2‐3‐fold and to stabilize their number until IL‐2 treatment withdrawal.^20^ Our data have demonstrated that the expansion of endogenous Tregs in mice receiving IL‐2c alone or in combination with adoptive transferred Tregs is similar, indicating that the frequency of Tregs is in strict relationship with the amount of IL‐2 available and is independent on the exogenous transfer of Tregs. This observation suggests that the combination of low‐dose IL‐2 therapy with adoptive transfer of polyclonal Tregs provides limited therapeutic benefit to the IL‐2 treatment alone. However, it should be noted that ex vivo polyclonal expansion, although it might not increase the frequency of Tregs with donor specificity, can provide additional functional properties to Tregs, which can add therapeutic improvement to the IL‐2 therapy, such as enhancing the expression of chemokine receptor to increase specific tissue trafficking and restoring or adding suppressive properties to endogenous Tregs that are often dysfunctional in disease patients.^23^

The combination of IL‐2c and adoptive transfer of donor‐specific Tregs significantly improves the therapeutic efficacy of both individual treatments in a model of skin transplantation. Phenotypic analysis of Tregs in recipient mice showed enhanced expression of the CTLA4 and inducible costimulator by donor‐specific Tregs, preferentially in the skin graft and draining lymph nodes, suggesting increased activation and suppressive function due to antigen recognition. The reported phenotypical changes induced by IL‐2 therapy on Tregs 15 were not significantly observed in the IL‐2c treated mice 10 days after skin transplantation due to the elapsed time from the last dose (day 3 after engraftment). Therefore, our data suggest that the improvement on graft survival in the combinational therapy is mainly related to the preferential expansion of donor‐specific Tregs by IL‐2c in graft‐associated tissues.

Our study further supports the importance of donor‐specific Tregs to regulate alloimmune responses and it shows limited effect of IL‐2 therapy by itself. However, the reduced frequency of allograft‐reactive Tregs early after transplantation and the short IL‐2 treatment provided in our model did not allow the sufficient expansion of endogenous donor‐specific Tregs. Therefore, we cannot exclude that IL‐2 therapy in combination with immunosuppressive drugs for prolonged periods of time could slowly augment the endogenous donor‐specific Treg pool by continuous antigen recognition. The application of new immunomonitoring approaches such as TCR repertoire analysis of donor‐reactive T cells could provide further information about the homeostatic characteristics of Tregs during IL‐2 therapy in clinical settings.^24^, ^25^

The fate of adoptive transferred Tregs in the clinic remains mostly unknown due to the suboptimal cell tracking methods currently available, limiting the assessment of cell survival, allograft trafficking and functional stability. It has been reported that tissue inflammation and other microenvironment cues such as the use of CNIs can compromise Treg homeostasis,^26^ and consequently, limit the survival and stability of adoptively transferred Tregs in nonhuman primates.^27^, ^28^ Therefore, complementary strategies to increase Treg expansion and secure their stability are indispensable in the transplantation setting, especially using donor‐specific Tregs, as the loss of their functional stability could lead to highly pathogenic responses to the allograft. Current reports from clinical trials using deuterium‐labelled Tregs during ex vivo expansion have demonstrated that transferred Tregs can be detectable up to 1 year in autoimmune and transplanted patients.^4^, ^5^ Although, similar to our results, the transferred cells proved to maintain the Treg phenotype in circulation, the percentage of Tregs remaining after 2 weeks was very little (<3% of total Tregs). Importantly, recent studies have reported that IL‐2 treatment can significantly expand and restore Treg dysfunction in the presence of CNIs treatment,^15^ and also maintain Treg stability in proinflammatory conditions.^29^ Therefore, the clinical utility of low‐dose IL‐2 combined with adoptive transfer of allospecific Tregs is particularly suited in transplantation settings where CNIs are the main IS drug.

We demonstrate here that IL‐2 treatment promotes a preferential expansion of adoptively transferred donor‐specific Tregs after skin transplantation leading to a synergistic effect in graft survival. However, the intrinsic restrains associated with the use of animal models limit the investigation of relevant immune responses important in a clinical setting. For instance, the BL/6.K^d^ into BL/6 model limits the analysis to Tregs with indirect allospecificity, because the graft does not present the allogenic major histocompatibility complex class II molecules required to prime Tregs through the direct allorecognition pathway. On the other hand, human transplantation generally involves high donor‐antigen mismatch and enhanced role of alloreactive memory T responses. Therefore, we believe that some form of T cell depletion will be essential, in combination with Treg therapy, to achieve transplantation tolerance in the clinic. Nevertheless, our data suggest that supplementing Treg administration with IL‐2 therapy will enhance their potency and increase the utility of this approach. Overall, this study provides novel understanding about Treg homeostasis during IL‐2 therapy and further highlights the advantage of donor specificity for Tregs immunoregulation in transplantation therapy.

## DISCLOSURE

The authors of this manuscript have no conflicts of interest to disclose as described by the *American Journal of Transplantation*.
