# Supplementary figures and images for: IL‐2 therapy preferentially expands adoptively transferred donor‐specific Tregs improving skin allograft survival
Source: Am J Transplant. 2019 Mar 15;19(7):2092–100. doi: 10.1111/ajt.15306 (PMC6618286; doi:10.1111/ajt.15306)

Figure S1

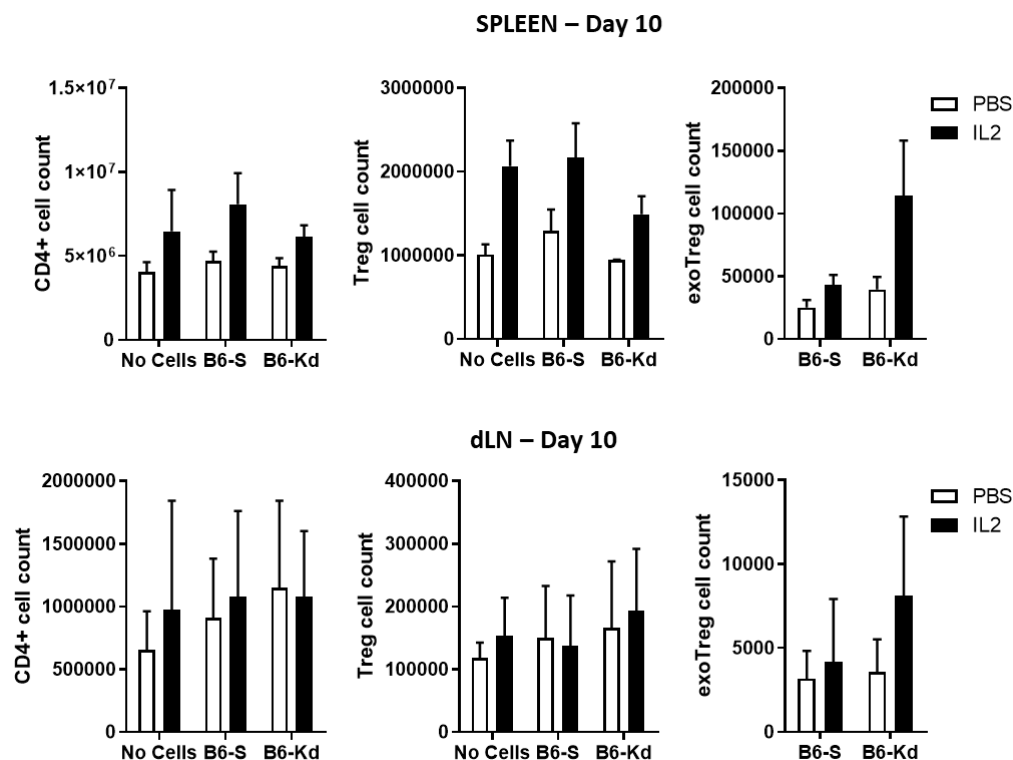

Figure S2

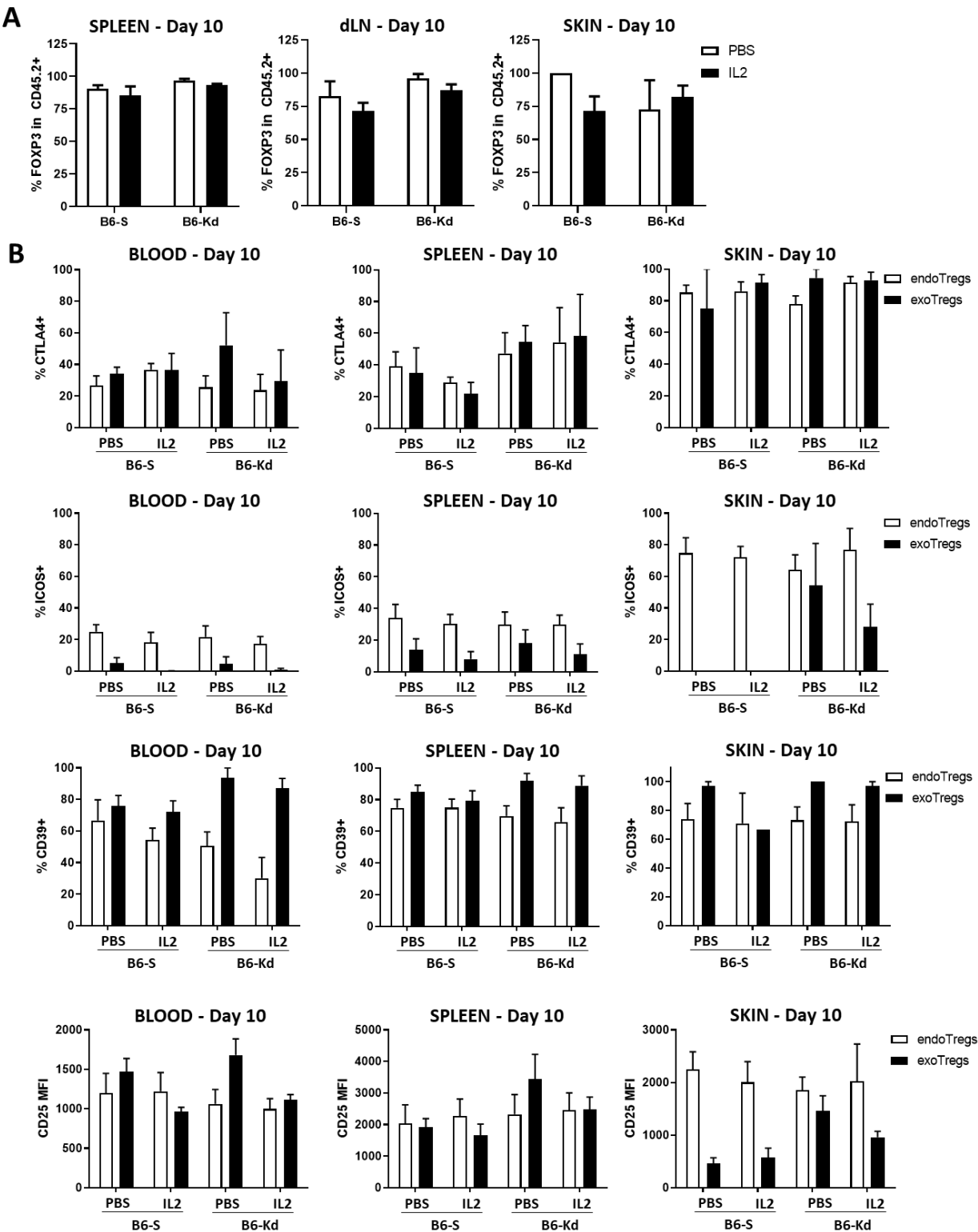

Supplement: Supplementary file 1 [file AJT-19-2092-s001.pdf]
