# Supplementary material for: IL‐2 therapy preferentially expands adoptively transferred donor‐specific Tregs improving skin allograft survival
Source: Am J Transplant. 2019 Mar 15;19(7):2092–100. doi: 10.1111/ajt.15306 (PMC6618286; doi:10.1111/ajt.15306)
Supplement: Supplementary file 2 [file AJT-19-2092-s002.docx]

**IL-2 therapy preferentially expands adoptively transferred donor-specific Tregs improving skin allograft survival**

Kulachelvy Ratnasothy, Jacintha Jacob, Sim Tung, Dominic Boardman, Robert Ian Lechler, Alberto Sanchez Fueyo, Marc Martinez-Llordella and Giovanna Lombardi.

**Supplemental Figure Legends**

Figure S1. Cellular quantification in different tissues after skin transplantation

Figure S2. Characterization of adoptive transferred Tregs in skin transplant model

**Supplemental Material and Methods**

*In vivo proliferation assay*: Cells were first labelled with 1µM carboxy-fluorescien diacetate succinimidyl ester (CFSE) for 15 min at 37^0^C. Then cells were washed twice with ice cold RPMI supplemented with 10% FCS. CFSE labelled Tregs (5x10^6^ cells) were injected either BL6 or B6 Kd host. Cell proliferation was assessed by Flowcytometry at day 3 after staining with CD4 APC.

*Generation of DC*: The DC were generated previously described (8). Briefly, bone marrow cells were treated with mixture of antibodies and unbound cells were selected using Dynal beads. Cells were cultured in the 10% FCS RPMI 1640 media in the presence of GM-CSF.

*T cell Proliferation (Suppression) Assays*: For In vitro polyclonal and antigen specific suppression assay, 5 x 10^4^ CFSE labelled CD4^+^T cells from BL6 and BL6RAG-/-TCR75 used as responders respectively. For both assay, T cell depleted splenocytes 1 X 10^5^ from BL6 CD45.1 congenic mice were used as stimulators in the presence of anti CD3 (1µg/ml) for polyclonal and Kd peptide (Kd 54-68) 1µg/ml for antigen specific suppression assay. Proliferation was assessed by Flow cytometry after 3 days of culture in the presence of different Treg cells in different ratio to T cells. Before performing flowcytometry, cells were harvested and stained for CD45.1 BV421 and CD4 APC for polyclonal suppression assay and CD45.1 BV421, CD90.1 APC (clone HIS51), CD4 PE Cy7 for antigen specific suppression assay. T cells depleted APC were gated out by identifying CD45.1 positive cells. Then CFSE stained cells gated from CD4 positive population with or without double positive to CD90.1 cells depending on assay.

*Flow cytometry*: Single cell suspensions were prepared by passing crushed spleen, Lymph nodes and skin (after incubating in 370C for 45min in the presence of Collagenase D 5mg/ml and DNase 0.01mU/ml in RPMI 1640 with 10%FCS media) in 70 µm cell filters. Following red blood cell lysis, cells were stained with fluorochrome. Anti CD45.1 BV421 (clone A20 -Biolegend) and Anti CD45.2 PE (clone 104 - Biolegend) were used to distinguish endogenous and exogenous origin. To assess the expansion of transferred cells, blood, spleen, lymph node skin were stained with anti CD45.1 BV421, anti CD45.2 PE, anti CD4 PECy7 (clone GK 1.5 – Biolegend), anti CD25 BV605 (clone PC61 – Biolegend), anti FoxP3 APC (clone FJK -16s – eBioscience) , and anti CD8 FITC (clone 53-6.7 – eBioscience) antibodies used as a panel. All stained cells were analysed on a Fortessa LSR flow cytometer at the Biomedical Research Centre and later data were analysed using FlowJo version 10.0.6.

**Supplemental Figure Legends**

Figure S1: Cellular quantification in different tissues after skin transplantation. Tregs from different tissues were analyzed 10 days after BL/6-K^d^ skin transplant to BL/6 mice receiving no cells, B6-S or B6-K^d^ Tregs in combination or not with IL-2c. Total cell counts from spleen (top panels) and draining lymph nodes (bottom panels) of CD4^+^ (left panels), total Tregs (middle panels) and exogenous Tregs (right panels). Results represent mean ± SEM of two independent experiments.

Figure S2: Characterization of adoptive transferred Tregs in skin transplant model. Tregs from different tissues were analyzed 10 days after BL/6-K^d^ skin transplant to BL/6 mice receiving no cells, B6-S or B6-K^d^ Tregs in combination or not with IL-2c. A) Frequency of FOXP3^+^ among transferred CD45.2^+^ cells in spleen draining lymph nodes and skin 10 days after skin transplant (right). B) Frequency of ICOS+, CTLA4+, CD39+ and CD25 MFI on endogenous (CD45.1) and exogenous transferred (CD45.2) Tregs in draining lymph nodes 10 days after transplantation. Results represent mean ± SEM of two independent experiments. * *P* < 0.05, ** *P* < 0.01, *** *P* < 0.001, **** *P* < 0.0001.
